# To: The Respiratory Rate-Oxygenation Index predicts failure of post-extubation high-flow nasal cannula therapy in intensive care unit patients: a retrospective cohort study

**DOI:** 10.5935/2965-2774.20230366-en

**Published:** 2023

**Authors:** Francesco Alessandri, Pierfrancesco Tozzi, Antonio Esquinas

**Affiliations:** 1 Department of General and Specialistic Surgery, Sapienza Università di Roma - Roma, Italy; 2 Azienda Ospedaliero Universitaria Policlinico Umberto I, Faculty of Medicine and Surgery, Sapienza Università di Roma - Roma, Italy; 3 Department of Intensive Care and Noninvasive Ventilatory Unit, Hospital Morales Meseguer - Murcia, Spain

## To the editor,

We have read with interest the study entitled “The respiratory rate-oxygenation index predicts failure of post extubation high-flow nasal cannula therapy in intensive care unit patients: a retrospective cohort study” published by Fuentes et al. in this journal.^(1)^ The authors proposed the respiratory rate-oxygenation (ROX) index as a predictor of post extubation high-flow nasal cannula (HFNC) therapy failure in pneumonia patients admitted to the intensive care unit. The ROX index is the ratio between oxygen saturation (SpO_2_) and fraction of inspired oxygen (FiO_2_) to respiratory rate. In patients suffering from pneumonia and acute hypoxic respiratory failure, it showed high accuracy in predicting HFNC failure 12 hours after treatment (ROC 0.74; 95%CI 0.64 - 0.84; p < 0.002), with a cutoff value < 4.88 to predict the need for tracheal intubation.^(2)^ In the study by Fuentes et al., a HFNC was placed after extubation to prevent respiratory failure, and the ROX index was calculated to predict the risk of reintubation. The ROX index was statistically lower in patients who had HFNC failure than in those who tolerated bridge therapy [median (IQR): 10.0 (7.7 - 14.4) *versus* 12.6 (10.1 - 15.6); p = 0.006] in terms of the ROX index to predict extubation failure, and the area under the ROC curve was 0.64 (95%CI: 0.53 - 0.75; p = 0.06).^(1)^ The application of this index has promise in quantifying the increase in the work of breathing imposed on these patients; however, we have some concerns. There is no universal agreement on the cutoff value of the ROX index, and in different studies, it varies from 2.7 to 9.2.^(3)^ This cutoff value has not been validated to predict HFNC failure in a different cohort of patients. In extubated patients, several factors can negatively affect the clinical course of the patient undergoing post extubation HFNC therapy, such as prolonged mechanical ventilation, severe neuropathy, and diaphragm and other respiratory muscle atrophy.^(4)^ Many studies have demonstrated that HFNC is superior to conventional oxygen therapy in reducing the incidence of treatment failure when used as an initial support strategy and that it reduces the rates of extubation failure.^(5)^ However, these patients are preconditioned by bridge therapy when there is no sign of respiratory failure or muscle fatigue. Perhaps these patients will develop respiratory failure at different times. Finally, we believe that the ROX index could be useful for evaluating the respiratory status of patients at the bedside; however, other prospective studies are needed to confirm the utility of this index in intensive care unit patients treated with HFNC as a bridge therapy and the optimal acquisition time of the ROX index.
